# Analysis of epidemiology and nomogram construction for prediction and clinical decision-making in gliomas

**DOI:** 10.3389/fimmu.2025.1624142

**Published:** 2025-08-01

**Authors:** Yuxin Zhao, Zihan Xu, Ying Liu, Ming Ye, Rui Chen, Zhongyu Cao, Hong Zhou, Yang Zhou

**Affiliations:** ^1^ Department of Ultrasound, Affiliated Hospital of Southwest Jiaotong University, The Third People’s Hospital of Chengdu, Chengdu, Sichuan, China; ^2^ Department of Medical Oncology, Cancer Center, West China Hospital, Sichuan University, Chengdu, Sichuan, China; ^3^ Lung Cancer Center, Cancer Center and State Key Laboratory of Biotherapy, West China Hospital of Sichuan University, Chengdu, Sichuan, China; ^4^ Institute for Breast Health Medicine, State Key Laboratory of Biotherapy, West China Hospital, Sichuan University, Chengdu, Sichuan, China

**Keywords:** gliomas, epidemiology, initial treatment, prognosis, nomogram

## Abstract

**Background:**

Gliomas are the most common primary malignant brain tumors with high mortality. Exploring the epidemiologic characteristics and prognostic factors of gliomas, and constructs a nomogram-based predictive model can help to evaluate the public health impact, optimize risk stratification, and guide treatment decision-making.

**Methods:**

This cross-sectional epidemiological analysis used the most recently released data from the Surveillance, Epidemiology, and End Results (SEER) database from January 1, 2000, to December 31, 2019. The SEER-18 database provided data for incidence, prevalence, survival, and initial treatment, as well as the establishment and validation of a nomogram to predict the survival probability of individual patients with gliomas.

**Results:**

Among 71,040 cases of glioma patients, the majority were male (40,500 [57.01%]) and White race (52,443 [73.82%]), with glioblastoma (41,125 [57.89%]) as the predominant histology type, primarily located at the cerebrum (49,307 [69.41%]), and mostly categorized as high-grade tumors (22,447 [31.60%]). The age-adjusted incidence rate of gliomas decreased from 4.42 per 100,000 persons in 2000 to 3.81 per 100,000 persons in 2019 [APC of -0.53 (95%CI, -0.71 to -0.34)]. In the incidence analysis among different tumor histology, grade and primary site, glioblastoma, high-grade tumor and primary site of cerebrum were with the highest incidence, respectively. Additionally, the incidence of different histology varied significantly among different age groups. In the multivariable analysis, age, histology, grade, site and treatment (chemotherapy, radiation and surgery) were identified as prognostic factors. Among these factors, age and grade had the most significant impact on prognosis. Furthermore, a predictive nomogram model for 1-/3-/5-year survival rates of gliomas was developed, incorporating the prognostic factors. For the training and test cohorts, the concordance indexes of the nomogram were 0.796 (95%CI, 0.792-0.805) and 0.799 (95%CI, 0.793-0.808), respectively.

**Conclusion:**

The incidence and survival of gliomas showed significant variations across different age, histology, grade, site, and treatment groups. The nomogram model based on these factors could accurately predict the survival among patients with gliomas and aid in optimizing treatment decisions.

## 1 Introduction

Gliomas are the most common primary malignant brain tumors and account for approximately 80% to 85% of malignant brain tumors in adults (1, 2). Gliomas consist of a heterogeneous group of tumors which originate from brain glial cells and diffusely infiltrate the brain parenchyma. According to the World Health Organization (WHO) classification, gliomas could be classified as low-grade (grade I, II) and high-grade gliomas (grade III, IV) based on histological features (3). Due to its’ high malignancy, mortality rate and recurrence risk, gliomas account for majority of deaths among patients with primary brain tumors (1).

Analyzing population-based trends offers valuable insights into the latest changes in incidence and mortality rates, which could influence strategies related to cancer prevention, monitoring, and treatment. However, many studies on the epidemiology and prognostic characteristics of glioma have been based on small samples or special population (4, 5). There are still no large sample studies for comprehensive epidemiological characteristics, treatment strategy and survival analysis of gliomas (such as different pathological types, sites of occurrence, grades or ages). Therefore, we conducted a population-based study utilizing data from the Surveillance, Epidemiology, and End Results (SEER) program to systematically analyze the epidemiologic, clinical, and prognostic characteristics of gliomas.

Assessing the prognosis of glioma patients remains challenging owing to the complex and heterogeneous nature of these tumors. Currently, the assessment methods for gliomas are limited and their predictive ability is poor. On the other hand, patients’ prognosis may also be linked to clinicopathologic characteristics, such as age, sex, race, and various other factors (6). Nomograms have emerged as a reliable and distinctive approach to prognosticate the outcomes of patients with cancer, effectively combining relevant prognostic factors to quantify the risk of mortality associated with malignant neoplasms (7, 8). Nevertheless, to the best of our knowledge, limited studies have utilized nomogram for prognosticating the outcomes of glioma patients. Therefore, the objective of this study was to construct a comprehensive nomogram, based on a sizable cohort of glioma patients from the SEER database, with the aim of accurately predicting 1-/3-/5-year overall survival (OS).

## 2 Materials and methods

### 2.1 Data source

The SEER database is a huge and public data source supported by the National Cancer Institute (NCI) in the United States, which covered information from 18 population-based cancer registries and representing 48% of the US population. Therefore, the SEER database can provide enormous amount of reliable information on cancer epidemiology and clinical characteristics. This study utilized data from the SEER database, which is publicly available and fully anonymized, without any access to personal identifiers or patient contact. Research using such de-identified secondary data is exempt from ethics committee review. Therefore, formal ethical approval was not required. Nonetheless, we conducted all analyses in strict adherence to ethical standards, ensuring confidentiality and integrity throughout the study process.

### 2.2 Data collection

**Figure 1:**
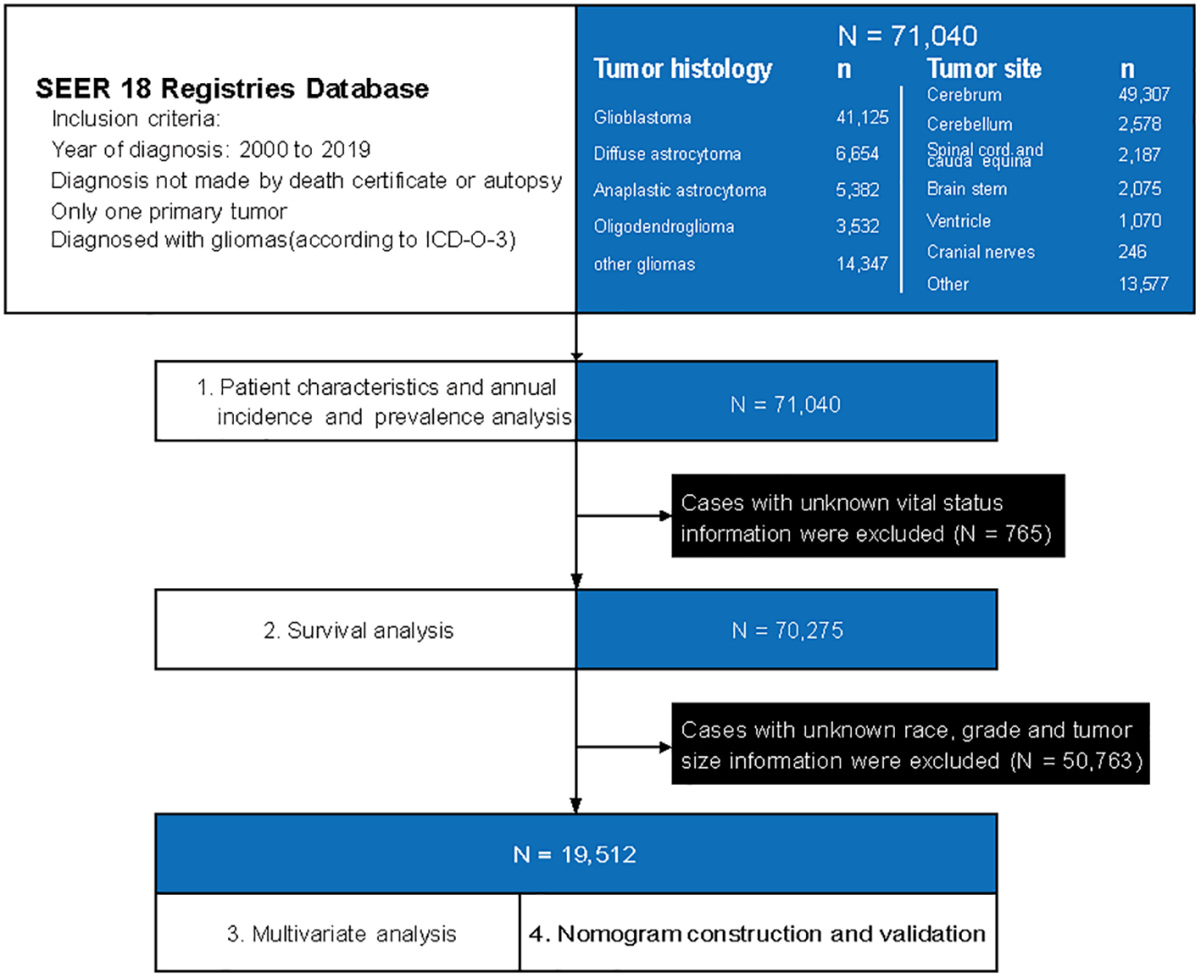
A flowchart of patient selection and study design. ICD-O-3, International Classification of Diseases for Oncology, Third Edition, and site codes.

We used histologic codes (**Supplementary Table 1**) from the *International Classification of Diseases for Oncology, Third Edition*, and site codes (ICD-O-3) (**Supplementary Table 2**) to identify patients who diagnosed with gliomas from January 1, 2000, to December 31, 2019. The correspondences between the codes and clinical/histological diagnoses were as follows: Anaplastic astrocytoma (9401/3), Diffuse astrocytoma (9400/3, 9410/3, 9411/3, 9420/3), Glioblastoma (9440/3, 9441/3, 9442/3), Oligodendroglioma (9450/3), Other gliomas (including Anaplastic oligodendroglioma: 9451/3, 9460/3; Ependymal tumors: 9391/3, 9392/3, 9393/3; Glioma malignant, not otherwise specified (NOS): 9380/3; Oligoastrocytic tumors: 9382/3; Pilocytic astrocytoma: 9421/1, 9425/3; Unique astrocytoma variants: 9381/3, 9424/3; Other neuroepithelial tumors: 9423/3, 9430/3). The design of the study is depicted in **Figure 1**. We extracted patient records from the SEER database, including basic clinical characteristics (such as age, sex, race and ethnicities, marital status, income, residence), tumor characteristics (such as histology type, tumor grade, tumor size and primary tumor site), and treatment information (chemotherapy, radiotherapy and surgery). Race and ethnicity were obtained through self-reporting. We categorized patients into five groups based on the following classification: Non-Hispanic White, Non-Hispanic Black, Non-Hispanic Asian and Pacific Islander, Non-Hispanic American Indian and Alaska Native, and Hispanic. The study followed the guidelines set forth by the Strengthening the Reporting of Observational Studies in Epidemiology (STROBE) for reporting observational studies.

### 2.3 Grade

The SEER classification scheme categorized tumor grades into four groups: grade I (GI), characterized by high discrimination; GII, indicating moderate differentiation; GIII, representing poor differentiation; and GIV, signifying undifferentiated or anaplastic cases. According to the grading criteria for gliomas, low-grade gliomas include GI and GII, while high-grade gliomas include GIII and GIV.

### 2.4 Nomogram establishment and verification

For multivariable analysis and development of the nomogram, we included only cases with complete information on key clinicopathologic variables, including age, sex, race, marital status, income, residence, tumor size, grade, primary tumor site, histologic subtype, and treatment details (surgery, radiation, chemotherapy). Cases with missing values for these variables were excluded from the modeling process, as these parameters were critical for multivariable analysis and risk estimation. The 19,512 eligible patients were divided into two groups according to the time of diagnosis: training group (2000-2010) and validation group (2011-2019). The nomogram was constructed based on prognostic factors determined by multivariable Cox proportional hazards regression models. The nomogram’s verification primarily relied on measures of internal (within the training cohort) and external (with the test cohort) discrimination and calibration and were tested by consistency index (C-index) and calibration curves. In addition, the accuracy of the nomogram in predicting 1-/3-/5-year survival was analyzed by the area under the receiver operating characteristic curve (AUC).

### 2.5 Statistical analysis

The statistical analysis was conducted between April 1 and May 30, 2024. Epidemiological analysis including age-adjusted incidence rates, limited-duration prevalence rates and annual percentage change (APC) were calculated based on age, sex, race, tumor grade, histology, and site using SEER*Stat software, version 8.3.8 (Surveillance Research Program, National Cancer Institute). Cancer-specific mortality, the outcome of interest, was obtained from the cause-specific death classification variable. The Kaplan–Meier method was used to estimate the 1-year, 3-year, and 5-year survival rates of patients with glioma. Cox proportional hazards multivariable regression analysis was employed to evaluate the association between clinical characteristic parameters with OS, by calculating hazard ratios (HRs) and 95% confidence intervals (CIs). Odds ratios (ORs) with 95% CIs were calculated using 2×2 contingency tables and the Wald method to compare baseline clinicopathologic characteristics between the training and validation cohorts, with the training group as the reference. Statistical analysis was performed using IBM SPSS Statistics version 23. All *P*-values were derived from two-sided tests, and statistical significance was defined as *P* < 0.05.

## 3 Results

### 3.1 Patient characteristics

During 2000 to 2019, a total of 71,040 patients diagnosed as gliomas were identified from the SEER database. Among them, the number of males (40,500 [57.01%]) is higher than that of females (30,540 [42.99%]). Younger patients (≤ 39 years, 17,911 [25.21%]) made up a larger proportion of patients with gliomas. Regarding to histology, glioblastoma (41,125 [57.89%]) was the most common histological type, followed by diffuse astrocytoma (6,654 [9.37%]). Anaplastic astrocytoma (5,382 [7.58%]) and oligodendroglioma (3,532 [4.97%]) accounted a smaller proportion. Among 28,037 patients with known grading information, 22,447 (80.06%) were high-grade gliomas and 5,590 (19.94%) were low-grade gliomas. Besides, Gliomas most commonly occur in the cerebrum, accounting for 49,307 cases (69.41%). The cerebellum, spinal cord and cauda equina, brain stem, ventricle, and cranial nerves accounted for 3.63% (2,578), 3.08% (2,187), 2.92% (2,075), 1.51% (1,070) and 0.35% (246), respectively (**Supplementary Table 3**).

### 3.2 Age at diagnosis

Among 27,964 patients with known age, sex, race, tumor site, histology, and grading information, we analyzed differences between age groups based on sex, race, tumor site and histology. Based on sex and race, there is no significant difference in the distribution of patients across different age groups. However, there are significant differences in the number of patients across different age groups when considering various tumor locations and histological types. Except for gliomas located in the cerebrum, which are evenly distributed across all age groups, tumors in other locations—such as the brainstem, cerebellum, spinal cord and cauda equina, and ventricles—are predominantly found in younger patients (≤ 39 years). Oligodendroglioma, diffuse astrocytoma, and anaplastic astrocytoma are the primary pathological types among younger patients (≤ 39 years), whereas glioblastoma is more common in older patients (**Table 1**).

**Table 1:** Age at diagnosis and tumor grade of glioma by sex, race, histology and primary tumor site.

Characteristic	Age at diagnosis (%)					Tumor grade (%)	
	≤ 39	40-49	50-59	60-69	≥ 70	Low-grade	High-grade
Sex							
Male	4243 (26.60%)	2537 (15.90%)	3356 (21.04%)	3147 (19.73%)	2671 (16.74%)	3018 (18.92%)	12936 (81.08%)
Female	3340 (27.81%)	1677 (13.96%)	2247 (18.71%)	2262 (18.83%)	2484 (20.68%)	2543 (21.17%)	9467 (78.83%)
Race							
Non-Hispanic White	4773 (23.17%)	2971 (14.42%)	4301 (20.87%)	4316 (20.95%)	4243 (20.59%)	3760 (18.25%)	16844 (81.75%)
Non-Hispanic Black	582 (35.02%)	273 (16.43%)	337 (20.28%)	268 (16.13%)	202 (12.15%)	408 (24.55%)	1254 (75.45%)
Non-Hispanic AI/AN	57 (41.30%)	28 (20.29%)	21 (15.22%)	18 (13.04%)	14 (10.14%)	48 (34.78%)	90 (65.22%)
Non-Hispanic Asian/PI	550 (36.96%)	256 (17.20%)	258 (17.34%)	219 (14.72%)	205 (13.78%)	314 (21.10%)	1174 (78.90%)
Hispanic	1621 (39.81%)	686 (16.85%)	686 (16.85%)	588 (14.44%)	491 (12.06%)	1031 (25.32%)	3041 (74.68%)
Primary tumor sites							
Brain stem	512 (71.01%)	71 (9.85%)	61 (8.46%)	52 (7.21%)	25 (3.47%)	326 (45.21%)	395 (54.79%)
Cerebellum	519 (69.57%)	53 (7.10%)	57 (7.64%)	53 (7.10%)	64 (8.58%)	449 (60.19%)	297 (39.81%)
Cerebrum	4840 (23.99%)	3220 (15.96%)	4223 (20.93%)	4051 (20.08%)	3839 (19.03%)	3227 (16.00%)	16946 (84.00%)
Spinal cord and cauda equina	330 (50.46%)	126 (19.27%)	99 (15.14%)	64 (9.79%)	35 (5.35%)	479 (73.24%)	175 (26.76%)
Ventricle	253 (66.58%)	39 (10.26%)	33 (8.68%)	33 (8.68%)	22 (5.79%)	184 (48.42%)	196 (51.58%)
Other brain and nervous system	1129 (21.34%)	705 (13.33%)	1130 (21.36%)	1156 (21.85%)	1170 (22.12%)	896 (16.94%)	4394 (83.06%)
Histology							
Anaplastic astrocytoma	1616 (33.55%)	804 (16.69%)	840 (17.44%)	796 (16.52%)	761 (15.80%)	0 (0.00%)	4817 (100.00%)
Diffuse astrocytoma	1368 (40.33%)	584 (17.22%)	545 (16.07%)	427 (12.59%)	468 (13.80%)	1909 (56.28%)	1483 (43.72%)
Glioblastoma	925 (7.35%)	1473 (11.71%)	3161 (25.13%)	3542 (28.15%)	3480 (27.66%)	110 (0.87%)	12471 (99.13%)
Oligodendroglioma	610 (46.71%)	354 (27.11%)	205 (15.70%)	90 (6.89%)	47 (3.60%)	1093 (83.69%)	213 (16.31%)
Other gliomas	3064 (52.22%)	999 (17.02%)	852 (14.52%)	554 (9.44%)	399 (6.80%)	2449 (41.73%)	3419 (58.27%)

Non−Hispanic AI/AN, Non-Hispanic American Indian/Alaska Native; Non−Hispanic Asian/PI, Non-Hispanic Asian or Pacific Islander.

### 3.3 Tumor grade

Next, in the analysis of differences between grade groups based on sex, race, tumor site and histology, we found that high-grade tumors were predominant in both male and female population and all race groups. Regarding site groups, high-grade tumor accounted a larger proportion in site of cerebrum, brain stem and ventricle, especially in cerebrum group (84.00%). However, low-grade tumors were predominantly located at site of cerebellum and spinal cord and cauda equina, especially in spinal cord and cauda equina group (73.24%). Pathological type is closely related to the malignancy of the tumor. For anaplastic astrocytoma and glioblastoma, nearly all cases are classified as high-grade due to their highly malignant biological behavior. In contrast, for less malignant pathological types, such as oligodendroglioma and diffuse astrocytoma, majority of cases are low-grade, with low-grade oligodendroglioma cases accounting for 83.69% (**Table 1**).

### 3.4 Annual incidence and prevalence based on basic clinical characteristics

**Figure 2:**
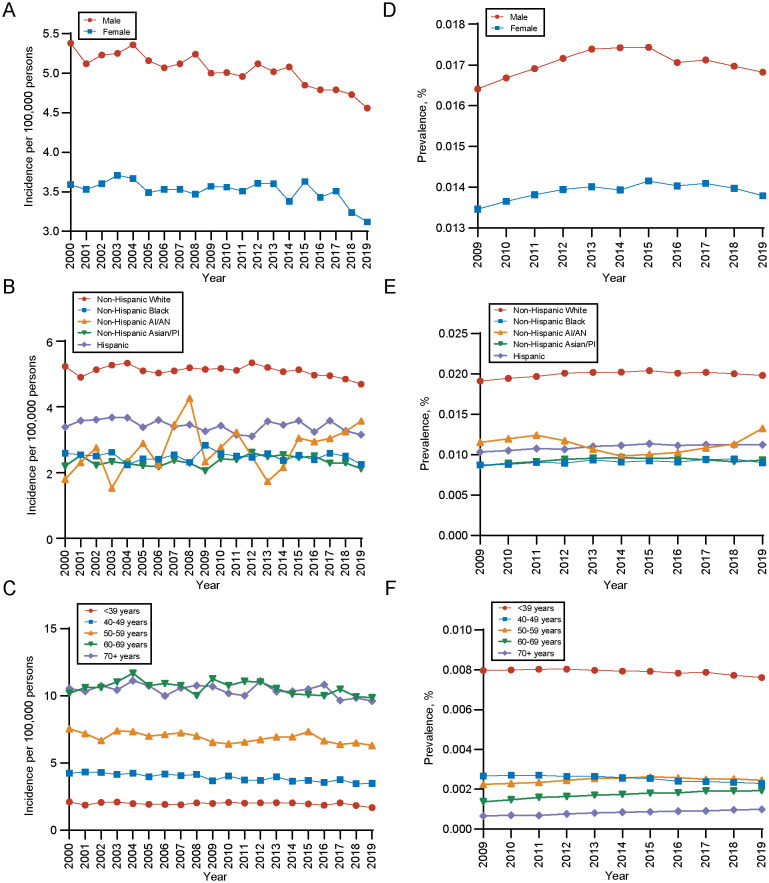
Incidence and 10-years limited prevalence of gliomas by sex, race and ethnicities, age at diagnosis. **(A)** Incidence of gliomas by sex. **(B)** Incidence of gliomas by race and ethnicities. **(C)** Incidence of gliomas by age at diagnosis. **(D)** 10-years limited prevalence of gliomas by sex. **(E)** 10-years limited prevalence of gliomas by race and ethnicities. **(F)** 10-years limited prevalence of gliomas by age at diagnosis. Non−Hispanic AI/AN, Non-Hispanic American Indian/Alaska Native; Non−Hispanic Asian/PI, Non-Hispanic Asian or Pacific Islander.

The overall age-adjusted incidence of gliomas was decreased from 4.42 per 100,000 persons in 2000 to 3.81 per 100,000 persons by 2019, with an APC of -0.53 (95%CI, -0.71 to -0.34), as presented in **Supplementary Figure 1A** (compared with all malignant neoplasms’ annual age-adjusted incidence (APC, -0.24; [95% CI, -0.39 to -0.09]). Detailed incidence information was in **Supplementary Table 4**. Regarding the sex of patients with gliomas, the age-adjusted incidence rates of male is higher than female. Over time, the incidence rates for both genders have shown a declining trend, with an APC of -0.64 (95% CI, -0.82 to -0.47) in males and -0.42 (95% CI, -0.69 to -0.15) in females (**Figure 2A**). Between different races, white populations had the highest incidence and experienced a decreased trend over years (APC, -0.27; 95%CI, -0.50 to -0.03), but the trend of gliomas among other races remains stable (**Figure 2B**). As for different age groups, the incidence of the population aged ≥ 70 years and those aged 60–69 years is highest among all age groups (**Figure 2C**). Detailed incidence information was in **Supplementary Table 5**.

In terms of the prevalence, the 10-year limited-duration prevalence of gliomas remained stable, changing from 0.01492% in 2009 to 0.01529% in 2019 (**Supplementary Figure 1B**, **Supplementary Table 6**). During the period, the prevalence of gliomas is significantly higher in male population than female population (**Figure 2D**) and white population had higher prevalence of gliomas than other races (**Figure 2E**), which was in line with the long-trend of incidence rates and patient characteristics. However, for different age groups, we found that population aged ≥ 70 years had the lowest prevalence of gliomas and population aged ≤ 39 years had the highest prevalence of gliomas (**Figure 2F**). This contrasts with the age-related incidence patterns, which could be attributed to the significant difference in mortality rates between these two age groups.

### 3.5 Incidence and prevalence based on tumor characteristics

**Figure 3:**
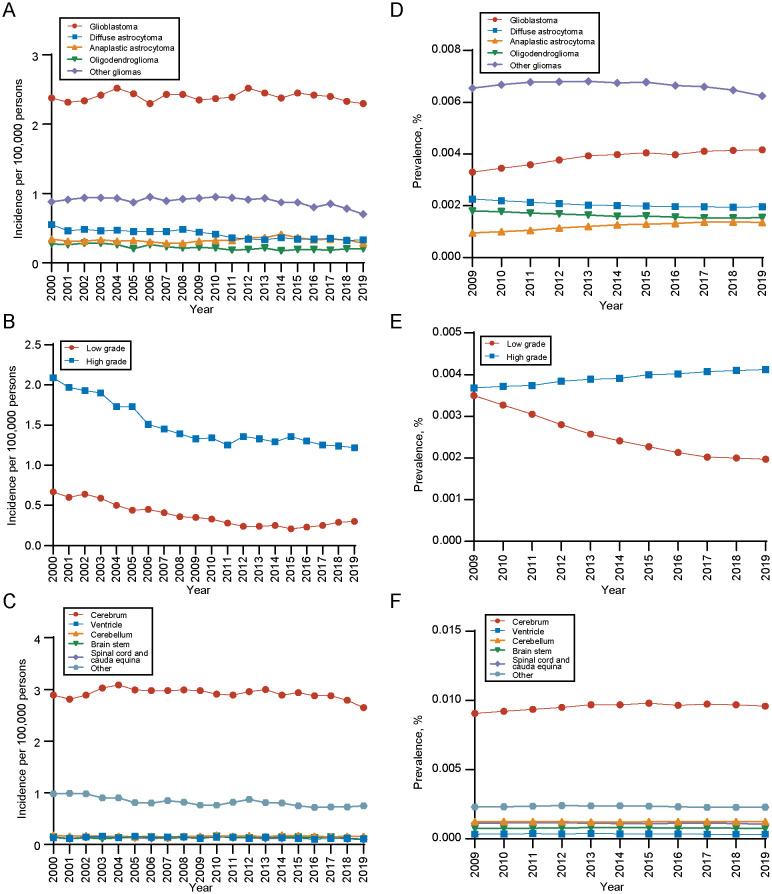
Incidence and 10-years limited prevalence of gliomas by tumor histology, grade and primary site. **(A)** Incidence of gliomas by tumor histology. **(B)** Incidence of gliomas by tumor grade. **(C)** Incidence of gliomas by primary tumor site. **(D)** 10-years limited prevalence of gliomas by tumor histology. **(E)** 10-years limited prevalence of gliomas by tumor grade. **(F)** 10-years limited prevalence of gliomas by primary tumor site.

During the analysis period, the overall incidence of gliomas is showing a declining trend, which is also reflected across different histological types, grades, and primary sites. As for different histology types, the incidence of glioblastoma was the highest. The incidence rate trend decreased in diffuse astrocytoma and oligodendroglioma, while stayed unchanged among other histology during the period (diffuse astrocytoma: APC, -2.56 [95% CI, -3.07 to -2.04]; oligodendroglioma: APC, -2.40 [95% CI, -3.10 to -1.70]) (**Figure 3A**). For different grades, the incidence of high-grade gliomas was significantly higher than that of low-grade, but the incidence trend of both decreased with time (high-grade gliomas, APC, -2.89; 95%CI, -3.49 to -2.28; low-grade gliomas, APC, -6.34; 95%CI, -7.32 to -5.36) (**Figure 3B**). In terms of the primary site, gliomas in cerebrum had the highest incidence rate (**Figure 3C**). Detailed incidence information was in **Supplementary Table 5**.

We also analyzed the 10-year limited-duration prevalence of gliomas according to tumor histology, grade and site. As for different histology type, the prevalence of glioblastoma was the highest, followed by diffuse astrocytoma (**Figure 3D**). In terms of tumor grade, the prevalence of high-grade gliomas was significantly higher than low-grade gliomas (**Figure 3E**). Among tumor sites, the prevalence of cerebrum was significantly higher than other sites (**Figure 3F**).

### 3.6 Incidence based on tumor histology, age and sex

**Figure 4:**
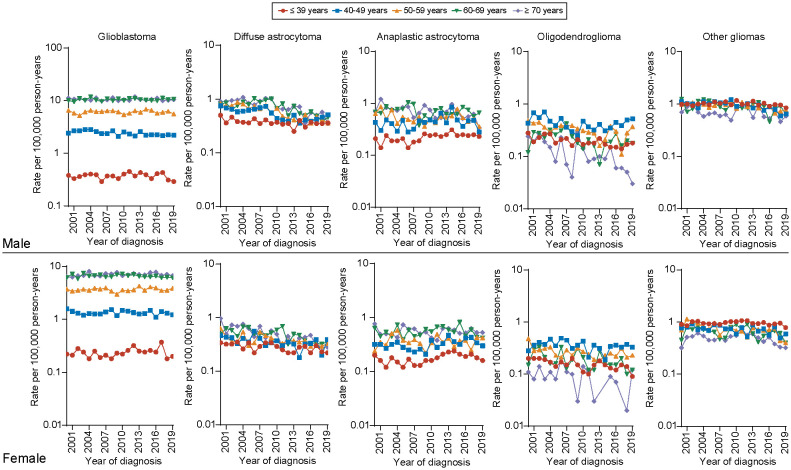
Incidence of gliomas by tumor histology, sex and age at diagnosis.

Furthermore, regarding the incidence of different histology types among all age groups, we observed that the incidence of glioblastoma was significantly higher in elder population (aged 60-69, ≥70 years) than younger population for both male and female population. The long-term incidence rate trend of glioblastoma decreased only for one group, male aged 40–49 years (APC, -1.14, [95% CI, -1.70 to -0.57]), but remained unchanged among other age groups. For diffuse astrocytoma, the incidence rate significantly decreases across all age groups for both males and females. The most pronounced declines are observed in males aged 50–59 years (APC, -4.49 [95% CI, -5.61 to -3.36]) and females aged ≥70 years (APC, -5.11 [95% CI, -6.66 to -3.53]). For anaplastic astrocytoma, the incidence rate remains higher in the elderly population (aged 60-69, ≥70 years) for both males and females. However, an increasing trend is observed only in the population aged ≤ 39 years (male: APC, 1.18 [95% CI, 0.64 to 3.13]; female: APC, 1.58 [95% CI, 0.05 to 3.12]), with no significant changes in other age groups. We also found that the incidence of low-grade gliomas as oligodendrogliomas, is higher in middle-aged individuals (aged 40-49,50–59 years) and shows a significant declining trend in older male and female patients (male aged ≥ 70 years: APC, -6.58 [95% CI, -10.18 to -2.84]; female aged 60–69 years: APC, -3.17 [95% CI, -5.92 to -0.34])) (**Figure 4**). Detailed incidence information was in **Supplementary Tables 7, 8**.

### 3.7 Survival

**Figure 5:**
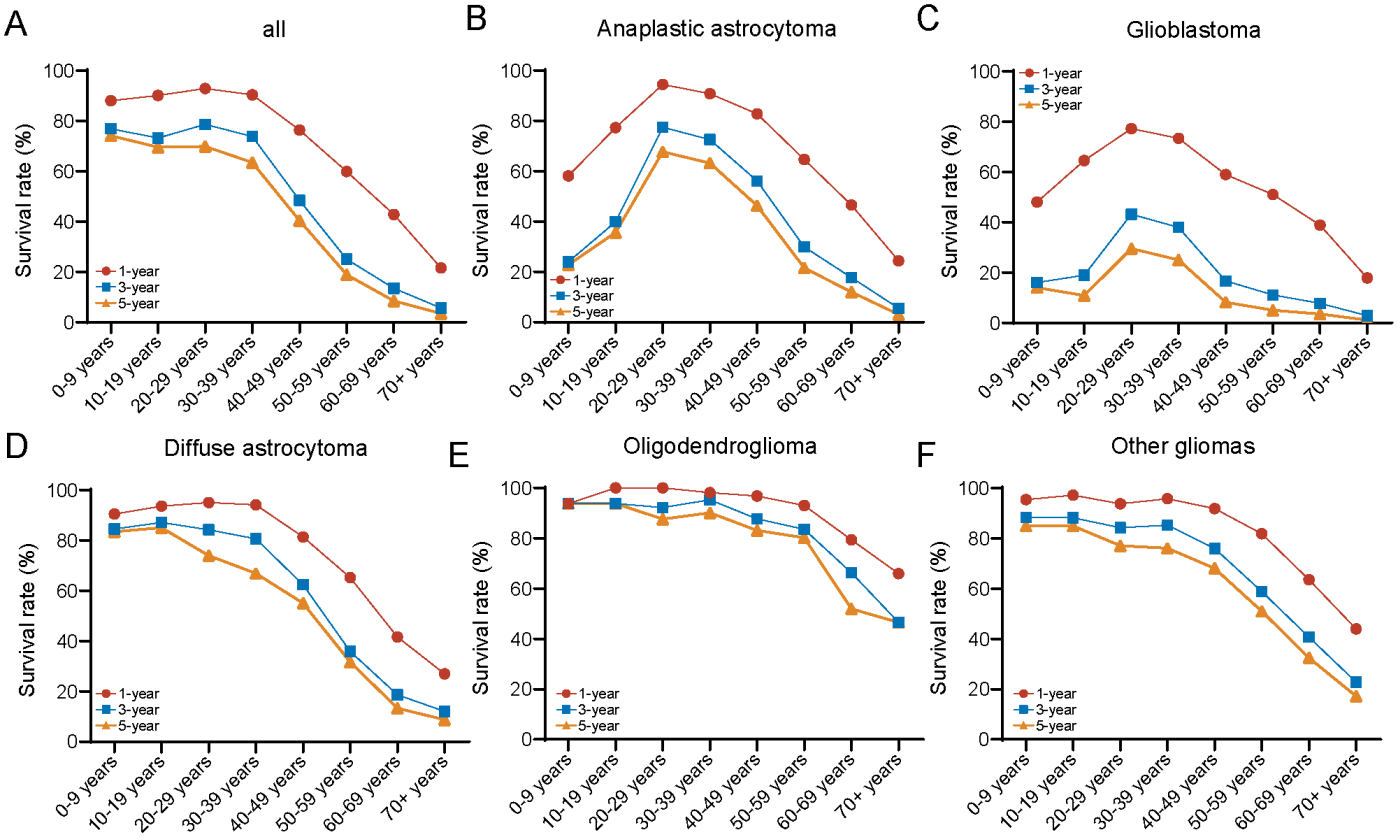
Comparison of 1-/3-/5-year relative survival for glioma by histology group. Age-specific 1-/3-/5-year relative survival for **(A)** all cases, **(B)** anaplastic astrocytoma, **(C)** glioblastoma, **(D)** diffuse astrocytoma, **(E)** oligodendroglioma, and **(F)** other gliomas.

Next, we analyzed the 1-, 3-, and 5-year survival rates across different age groups and plotted the results as a line graph. Each point on the curve represents the proportion of patients within that age group who survived at least 1 year, at least 3 years, and at least 5 years, respectively. For the overall population, younger patients (≤ 39 years) exhibit higher 1-, 3-, and 5-year survival rates. In contrast, for older adult patients (≥ 40 years), the 1-, 3-, and 5-year survival rates decline rapidly with increasing age (**Figure 5A**). The survival rates across different histopathological subtypes show varying characteristics as age progresses. For the more aggressive pathological subtypes, such as glioblastoma and anaplastic astrocytoma, the 1-, 3-, and 5-year survival rates are generally low, displaying an inverted “V” pattern. Specifically, patients with glioblastoma and anaplastic astrocytoma have the best 1-, 3-, and 5-year survival rates in the 20–39 age range, while those younger or older than this age group show poorer survival outcomes (**Figures 5B, C**). For diffuse astrocytoma, the survival rate trend with age is similar with that of the overall population (**Figure 5D**). In the case of oligodendrogliomas, the prognosis is best, with consistently high 1-, 3-, and 5-year survival rates across most age groups (0–59 years), but a marked decline in survival is observed in patients over ≥ 60 years (**Figure 5E**). For other histological types of gliomas, the age-related trends in survival rates were similar with those observed in the overall population; however, the 1-, 3-, and 5-year survival rates were significantly better (**Figure 5F**). Between genders, the 1-, 3-, and 5-year survival rates for males and females follow trends similar with those of the overall population, with males exhibiting slightly worse long-term survival (3- and 5-year) compared to females (**Supplementary Figures 2A, B**). Regarding different grades, high-grade gliomas show significantly poorer survival, also displaying a distinctive inverted “V” pattern (**Supplementary Figures 2C, D**). The risk tables were showing in **Supplementary Materials** (**Supplementary Table 9**).

### 3.8 Multivariable analysis of OS

**Figure 6:**
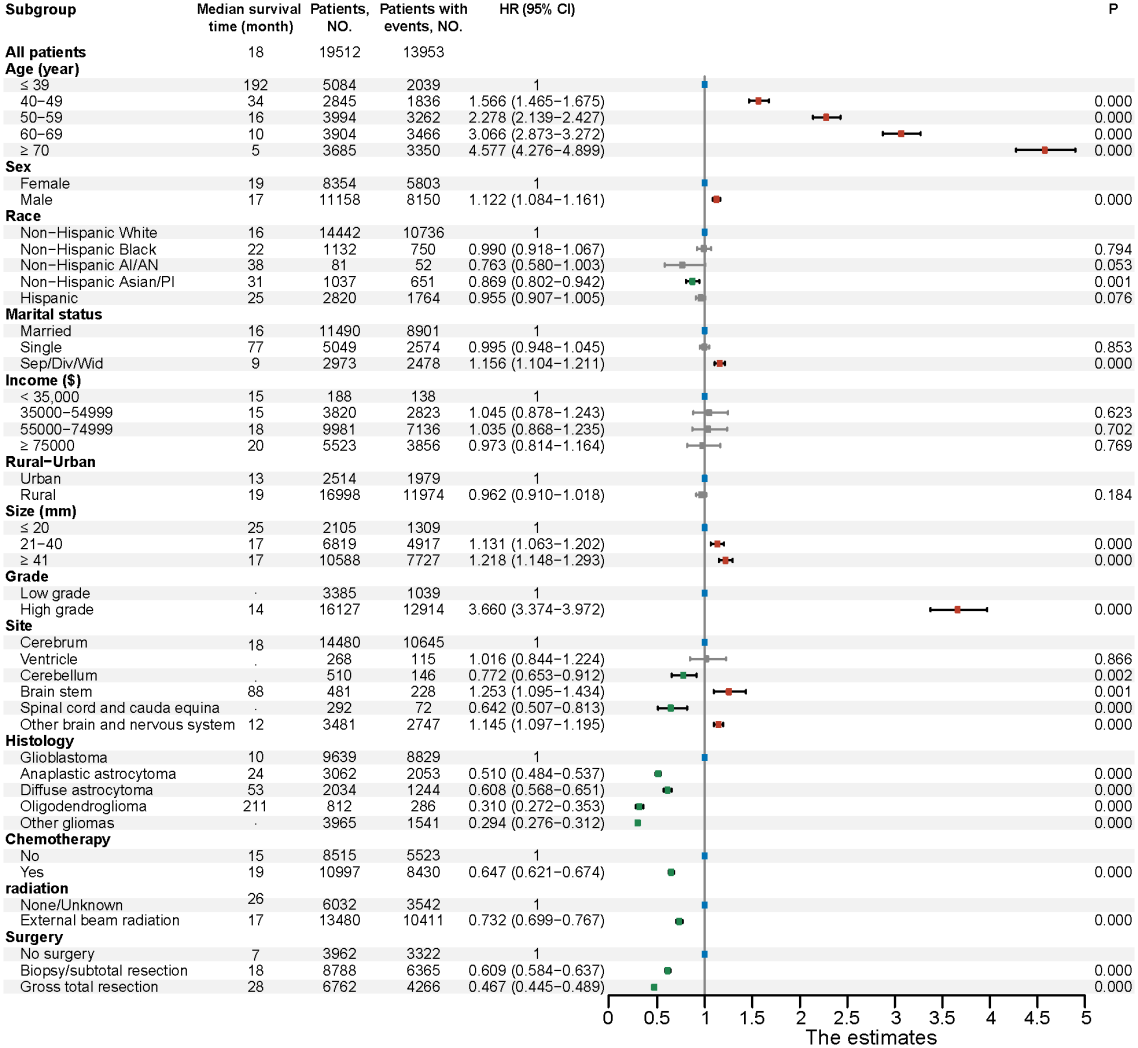
Multivariable regression analysis for gliomas. HR, Hazard ratio; CI, Confidence interval; Non−Hispanic AI/AN, Non-Hispanic American Indian/Alaska Native; Non−Hispanic Asian/PI, Non-Hispanic Asian or Pacific Islander; Sep/Div/Wid, Separated/Divorced/Widowed.

Then, we display a forest plot summarizing the results of the multivariate Cox proportional hazards regression analysis. Each row represents a prognostic factor or subgroup, with the dot indicating the HR and the horizontal line showing the 95% CI. An HR greater than 1 suggests a higher risk of death compared to the reference group, while an HR less than 1 indicates a lower risk. If the CI does not cross 1, the association is considered statistically significant. Corresponding p-values are provided to assess the significance of each factor. In addition, the median survival time (in months) for each subgroup is shown to aid interpretation. Among the 19,512 patients with complete information on age, sex, race, marital status, income, residence, tumor size, grade, primary tumor site, histology, and treatment (including chemotherapy, radiation, and surgery), these variables were included as potential prognostic factors in the multivariable model. We found that age is a significant independent factor affecting prognosis, with the risk of mortality increasing markedly as age advances. Glioma patients aged ≥ 70 years have the highest risk of death (HR, 4.58; 95% CI, 4.28-4.90). As for tumor grade, our study revealed that high-grade gliomas (HR, 3.66; 95% CI, 3.37-3.97) was significant risk factor for patient prognosis compared with low-grade gliomas. In terms of histology type, we observed that patients with glioblastoma had the worst prognosis. Take it as reference, oligodendroglioma had the best prognosis (HR, 0.31; 95% CI, 0.27-0.35), followed by anaplastic astrocytoma (HR, 0.51; 95% CI, 0.48-0.54), diffuse astrocytoma (HR, 0.61; 95% CI, 0.57-0.65). Between different primary tumor sites, with reference to patients with gliomas in cerebrum, patients with gliomas in brain stem (HR, 1.25; 95% CI, 1.10-1.43) had the worst prognosis. On the contrary, patients with gliomas in spinal cord and cauda equina (HR, 0.64; 95% CI, 0.51-0.81) had the best prognosis. Regarding treatment, whether it be chemotherapy, radiotherapy, or surgery, all serve as favorable protective factors for glioma patients. Notably, patients who undergo gross total resection have a significantly reduced risk of death (HR, 0.47; 95% CI, 0.45-0.49). Besides, other parameters, including sex, race and ethnicity, marital status, and tumor size were found to be slightly associated with prognosis (**Figure 6**).

### 3.9 Nomogram

**Figure 7:**
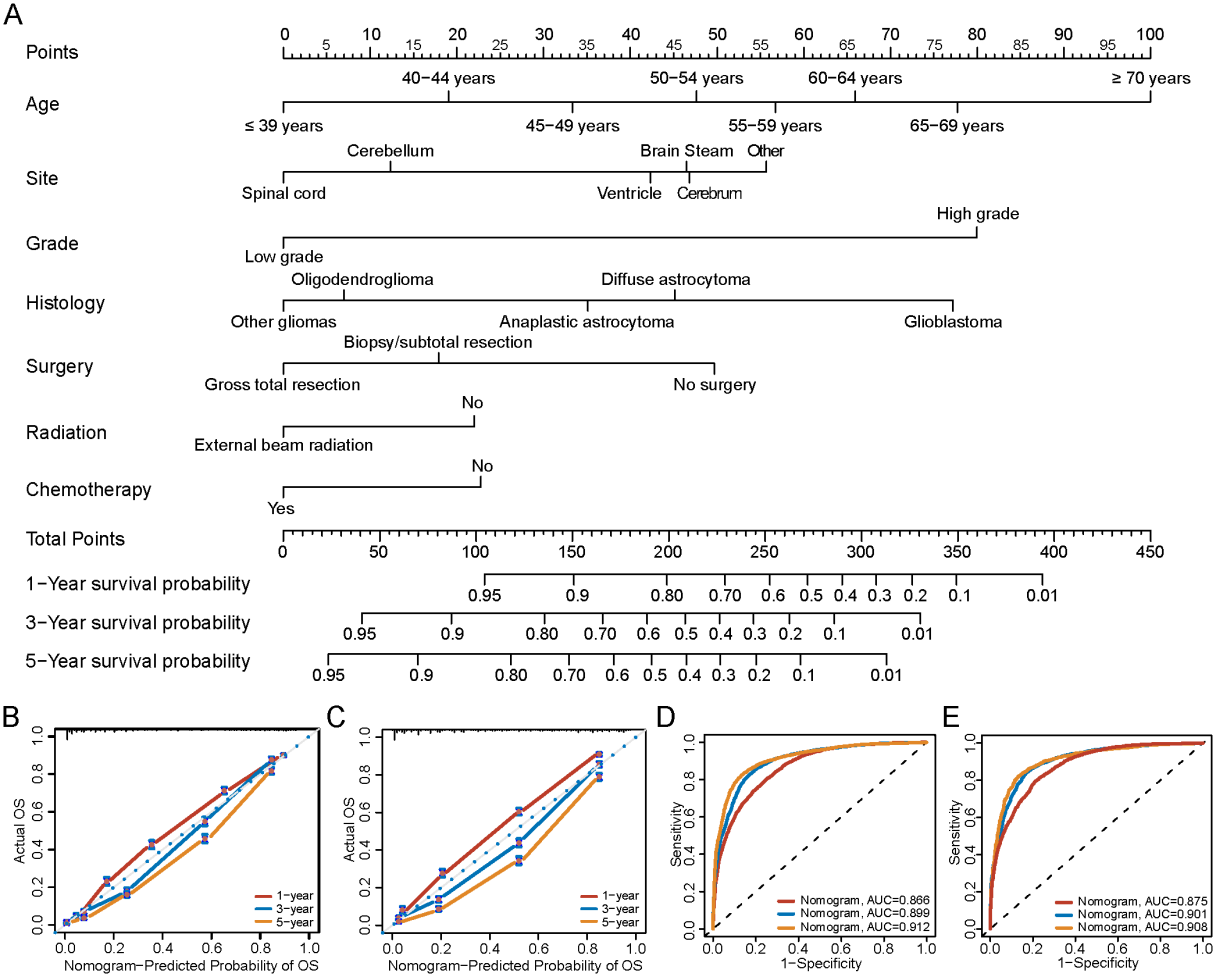
Nomogram to predict the 1-/3-/5-year survival probabilities of patients with gliomas. **(A)** Points of age, histology, grade, primary site and therapy (chemotherapy, radiation and surgery) are obtained by drawing a line upward from the corresponding values to the “Points” line. The sum of the points of these 7 factors is located on the “Total points” line, and a line projected down to the bottom scales determines the probabilities of 1-/3-/5-year survival probabilities. **(B)** Calibration plots of the nomogram for 1-/3-/5-year survival probabilities in the training set, and **(C)** the validation set. The gray line represents the ideal nomogram. The predicted probability of OS by the nomogram is projected onto the x-axis, and the actual OS is projected onto the y-axis. Error bars indicate 95% CIs. **(D)** AUC of nomogram for predict 1-/3-/5-year survival in training group, and **(E)** validation group. OS, Overall survival; AUC, the area under the receiver operating characteristic curve.

Based on the 19,512 eligible patients included in the multivariable analysis, we developed and validated the nomogram by dividing the cohort into a training group (diagnosed between 2000–2010) and a validation group (diagnosed between 2011–2019). The baseline clinicopathologic characteristics of both groups, along with ORs and corresponding 95% CIs using the training group as the reference, are summarized in **Supplementary Table 10**. Baseline clinicopathologic characteristics and associated survival metrics—including mean time to death, standard error of time to death, median time to death, and conditional mean survival times for patients who survived at least 1 year, 3 years, and 5 years—are presented in **Table 2**. In the training group, a nomogram model was established based on factors associated with OS, which were screened out by Cox proportional hazards regression model (**Figure 7A**). Each numerical value or category of variables comprised in the nomogram were relevant to a specific score on the “Point” scale. By adding up all individual scores and finding the corresponding point on the “Total Points” scale, a vertical line could then be drawn downwards to intersect with the “1-/3-/5-year survival probability” scale, thereby revealing the respective survival probability. Age had the greatest significance, contributing a maximum of 100 points. Tumor grade (80 points), histology (77 points), primary tumor site (56 points), surgery (50 points), chemotherapy (23 points), and radiation (22 points) were also individually associated with OS. **Supplementary Table 11** shows the specific scores for each variable and the estimated 1-, 3-, and 5-year survival probabilities corresponding to a wide range of total point values. For the verification of the nomogram, the C-indexes of OS prediction in the nomogram were 0.796 (95%CI, 0.792-0.805) in the training cohorts and 0.799 (95%CI, 0.793-0.808) in the test cohorts. Meanwhile, the calibration plots demonstrated a strong alignment between nomogram-predicted outcomes and actual results in both the training and validation cohorts (**Figures 7B, C**). Additionally, the survival prediction ability of the nomogram was evaluated using ROC curves. The AUC for predicting 1-, 3-, and 5-year survival rates in the training cohort was 0.866, 0.899, and 0.912, respectively (**Figure 7D**). In the validation cohort, the AUC for predicting 1-, 3-, and 5-year survival rates was 0.875, 0.901, and 0.908, respectively (**Figure 7E**), demonstrating excellent predictive accuracy.

**Table 2:** Baseline clinicopathological characteristics and survival metrics of patients in the nomogram construction.

Variables	Patients (n=19512) N (%)	Mean time to death (month)	Standard error	Median of time to death (month)	Conditional mean of time to death (month)		
					1-year	3-year	5-year
Age, y							
≤ 39	5084 (26.06%)	146.94	1.526	192.00	161.52	189.31	204.91
40-49	2845 (14.58%)	89.91	1.962	34.00	115.85	170.04	194.76
50-59	3994 (20.47%)	45.94	1.259	16.00	72.78	145.17	177.93
60-69	3904 (20.01%)	24.43	0.827	10.00	50.04	114.78	155.35
≥ 70	3685 (18.89%)	12.75	0.569	5.00	43.78	110.35	152.13
Sex							
Male	11158 (57.19%)	65.61	0.923	17.00	106.13	168.60	193.52
Female	8354 (42.81%)	75.07	1.127	19.00	121.61	179.35	202.32
Race							
Non-Hispanic White	14442 (74.02%)	64.51	0.801	16.00	108.13	171.87	196.23
Non-Hispanic Black	1132 (5.80%)	79.52	3.147	22.00	123.90	178.57	199.36
Non-Hispanic AI/AN	81 (0.42%)	82.34	10.838	38.00	117.22	151.37	175.93
Non-Hispanic Asian/PI	1037 (5.31%)	86.59	3.382	31.00	119.16	170.27	203.31
Hispanic	2820 (14.45%)	86.43	2.079	25.00	127.71	180.35	201.12
Marital status							
Single	5049 (25.88%)	118.58	1.609	77.00	153.08	193.79	210.12
Married	11490 (58.89%)	56.16	0.838	16.00	93.65	158.76	187.46
Sep/Div/Wid	2973 (15.24%)	38.32	1.408	9.00	87.78	157.91	185.55
Household income							
< $35,000	188 (0.96%)	52.92	5.957	15.00	92.10	154.19	178.20
$35,000-$54,999	3820 (19.58%)	63.16	1.578	15.00	109.63	174.77	198.93
$55,000-$74,999	9981 (51.15%)	70.43	1.000	18.000	114.60	173.51	197.82
≥ $75,000	5523 (28.31%)	73.12	1.367	20.00	111.86	172.52	196.04
Urban/rural							
Rural	2514 (12.88%)	53.60	1.770	13.00	99.42	165.86	192.14
Urban	16998 (87.12%)	72.10	0.777	19.00	114.54	174.44	198.28
Size (mm)							
≤ 20	2105 (10.79%)	93.27	2.400	25.00	133.85	194.99	215.36
21-40	6819 (34.95%)	68.56	1.205	17.00	111.86	179.85	203.97
≥ 41	10588 (54.26%)	65.50	0.945	17.00	108.21	163.81	188.36
Grade							
Low-grade	3385 (17.35%)	171.44	1.704	NR	183.36	198.72	208.19
High-grade	16127 (82.65%)	47.05	0.666	14.00	84.44	152.47	185.48
Primary tumor sites							
Brain stem	481 (2.47%)	127.56	5.271	88.00	165.80	216.90	226.72
Cerebellum	510 (2.61%)	171.43	4.632	NR	201.88	225.53	233.80
Cerebrum	14480 (74.21%)	64.74	0.800	18.000	103.78	164.85	190.06
Spinal cord and cauda equina	292 (1.50%)	182.26	5.772	NR	204.13	222.78	229.07
Ventricle	268 (1.37%)	136.35	7.006	NR	174.81	212.74	220.53
Other brain and nervous system	3481 (17.84%)	51.72	1.498	12.00	101.88	164.35	194.12
Histology							
Anaplastic astrocytoma	3062 (15.69%)	75.16	1.909	24.00	109.98	163.80	188.01
Diffuse astrocytoma	2034 (10.42%)	98.96	2.357	53.00	135.51	170.39	188.06
Glioblastoma	9639 (49.40%)	19.35	0.419	10.00	39.76	97.52	147.00
Oligodendroglioma	812 (4.16%)	166.51	3.325	211.00	174.96	188.00	195.70
Other gliomas	3965 (20.32%)	148.32	1.764	NR	170.01	198.69	213.74
Chemotherapy							
No	8515 (43.64%)	83.71	1.184	15.00	153.85	192.04	205.81
Yes	10997 (56.36%)	58.47	0.868	19.00	86.31	152.99	185.57
Radiation							
None/Unknown	6032 (30.91%)	97.62	1.476	26.00	172.40	198.67	210.53
External beam radiation	13480 (69.09%)	57.09	0.773	17.00	88.48	154.91	185.55
Surgery							
No surgery	3962 (20.31%)	39.01	1.239	7.00	96.43	164.36	189.22
Biopsy/subtotal resection	8788 (45.04%)	67.02	1.044	18.00	107.70	167.13	191.45
Gross total resection	6762 (34.66%)	90.98	1.317	28.00	123.43	182.52	206.02

Non−Hispanic AI/AN, Non-Hispanic American Indian/Alaska Native; Non−Hispanic Asian/PI, Non-Hispanic Asian or Pacific Islander; Sep/Div/Wid, Separated/Divorced/Widowed.

## 4 Discussion

We conducted a retrospective study by integrating a relatively large amount of data in the SEER program to evaluate the epidemiologic and prognostic factors for patients diagnosed with gliomas from 2000 to 2019. Regarding the patient characteristics, we found that gliomas predominantly occurred in males and white population, which was consistent with the previous studies (9, 10). The underlying reasons might be due to the genetic susceptibilities, environmental exposures, or a combination of these factors (11). Several studies have demonstrated that the incidence of cancer increased with age (12, 13). In our research, we also found that elder population had the highest incidence but lowest prevalence of gliomas, which indicated that elder population had poorer survival than younger population. Besides, be in line with recent research (14), glioblastoma, classified as high-grade glioma, was the most common histological type of glioma with the highest incidence rate in our research. We further discovered that the incidence of different histology types was associated with patient’s age. High-grade gliomas as glioblastoma were more prevalent in the elderly population, while low-grade gliomas as oligodendroglioma tended to occur more frequently in middle-age population, which were also confirmed in previous studies (15, 16).

We observed a mild decrease trend of the overall incidence of gliomas during the analysis period, which is consistent with recent researches (4, 17). The decreased incidence trend might be relevant to the decreased incidence of low-grade gliomas as oligodendroglioma and diffuse astrocytoma. However, high-grade gliomas, such as glioblastoma, also keep a highest incidence rate and remained stable during the period. The incidence of gliomas fluctuated due to a variety of factors. The observed decline may be attributed to the 2016 WHO CNS reclassification, which redefined certain tumor entities—such as reassigning IDH-mutant gliomas and excluding non-diffuse gliomas—thereby reducing overdiagnosis and altering histologic coding practices in cancer registries (17). Additionally, the advancement of imaging technologies and diagnostic procedures could lead to more accurate diagnoses of gliomas, this would help to eliminate overdiagnosis or misdiagnosis (18), thus reducing the reported incidence rate. On the other hand, many studies consistently suggesting that atopic diseases, such as allergies asthma, could reduce the risk of glioma, which may be due to the increasing immune monitoring of the overactive immune state (19).

In this research, we carried out the survival analysis, which allowed us to validate the importance of the related clinical parameters. In the further performed multivariable survival analysis using the Cox proportional hazards regression model, we found that patients age was the most significant risk factor for prognosis, which was consistent with prior studies (20, 21). The mechanism of age-related decreased prognosis might be related to the decline of the tolerance to the treatment and negatively impacts on the immune system (11, 22). On the other hand, we believed that high-grade gliomas as glioblastoma had a significantly higher incidence rate in elder population also contributed to the poorer prognosis. Besides, the prognosis of high-grade gliomas was significantly poorer than low-grade gliomas according to our study, which was also be certified in other studies (23). This might be related to high-grade gliomas with high expression of O6-methylguanine-DNA-methyltransferase promoter methylation, 1p19q co-deletion, isocitrate dehydrogenase gene mutations (24). In our study, histological type was the tertiary significant risk factor affecting the prognosis of glioma. Many studies have reported the poor prognosis of glioblastoma with a median survival of only 15 months despite advances in surgical resection, radiotherapy and chemotherapy (25). Researches indicated that high expression of CD44 (26) and lower expression of CNTN3 (27) were related to the poor prognosis of glioblastoma. Besides, primary tumor site was also risk factor related to prognosis, gliomas located at the brain stem had the worst outcome compared with other tumor sites in our research, which might be due to the primary site of glioma was associated with surgical option and postoperative mortality rate (28).

Previous studies have explored the utilization of nomogram in cancers (20, 29). In our research, we extracted a large amount of glioma cases to constructed a nomogram model. To our knowledge, our research was the first research that established a nomogram model apply to gliomas patients with the largest sample size until now. After multivariable survival analysis, our model comprised seven significant prognostic variables (age, site, histology, grade, chemotherapy, radiation and surgery), which could predict 1-/3-/5-year survival rates of glioma patients simply and accurately. Besides, our results demonstrated that the nomogram showed better predictive ability than the other clinical parameters. In conclusion, this efficient tool could more simply and accurately predict the prognosis of gliomas by evaluating various variables, indicating good clinical applicability of assisting clinical decision-making.

This study utilized the CBTRUS histologic classification based on ICD-O-3 codes, as provided by the SEER database. Given that our analysis included cases diagnosed between 2000 and 2019—prior to the release of the WHO CNS5 (2021) classification—molecular data such as IDH mutation and 1p/19q codeletion were not available (30). Therefore, applying the WHO CNS5 system was not feasible. The CBTRUS system remains widely used in population-based studies and allows for consistent comparison across large datasets, as demonstrated in prior SEER-based research (31, 32). However, it lacks the molecular resolution of WHO CNS5, which may limit biological precision. For example, glioblastoma under CBTRUS includes both IDH-wildtype and IDH-mutant tumors, despite differing prognoses. While appropriate for epidemiologic trends and survival modeling in the SEER context, our findings should be interpreted with caution, and future studies integrating molecular markers are warranted to align with modern diagnostic standards.

We acknowledge that our study did not include molecular epidemiology analyses, such as IDH mutation-specific trends (33, 34). This limitation stems from the inherent constraints of the SEER database, which does not contain IDH mutation, 1p/19q codeletion, or other key molecular markers required for WHO CNS5 (2021) classification (3). Moreover, our dataset spans cases diagnosed from 2000 to 2019—a period preceding the routine clinical implementation of molecular testing and the publication of the WHO CNS5 classification. In contrast, Ostrom et al. (35) utilized multi-institutional or integrated molecular-pathology datasets with more recent case inclusion, allowing for subtype-specific incidence analysis (e.g., IDH-mutant vs. IDH-wildtype gliomas). While such studies offer greater biological precision, they often rely on smaller cohorts and selected populations, whereas our study leverages SEER’s large-scale, population-based design to examine long-term trends across >71,000 glioma cases. This provides robust epidemiological insights into incidence, treatment, and survival patterns over two decades, albeit without molecular stratification. We fully agree that the integration of molecular features is essential for future glioma epidemiology. As molecular profiling becomes standard in clinical practice and registry systems evolve to capture such data, future SEER-based studies will be able to address IDH-specific trends with greater accuracy. Until then, CBTRUS histologies provide a valuable, if limited, framework for population-level glioma research.

Nevertheless, our study also exits some limitations. First, the SEER database did not include all cancer patients in the United States, which may have biased the incidence and prevalence estimates in this study. Second, there was lack of the information about the functional status and therapy strategies in patients with gliomas, which might be associated with the prognosis of these patients. Additionally, the nomogram developed in this study does not include molecular markers such as IDH mutation status, which are now recognized as important predictors of immunotherapy response. IDH-mutant and wild-type gliomas differ in their immune landscapes and treatment sensitivity. As our model is based on historical SEER data lacking molecular and immunotherapy-related variables, its applicability to current clinical practice may be limited. Future models should incorporate molecular and immunologic factors to improve personalized prognostication. However, our study also has several strengths. To the best of our knowledge, this study is one of the largest and most up-to-date studies on glioma, including all pathological types and age groups. Its size and long-term follow-up data largely fill the gap and provide comprehensive epidemiological and survival data on glioma.

Overall, the incidence of gliomas was decreased during 2000-2019. Specially, the incidence and prevalence of glioma showed significant variations across different age groups, pathology, tumor locations, and grade. In addition, the prognosis analysis revealed that elder age, sex, tumor grade, tumor histology, primary tumor site and treatment strategy were risk factors associated with prognosis. Furthermore, a nomogram constituted and validated in this study, may accurately predict the 1-/3-/5- year survival of patients with gliomas and help to optimize clinical decision-making.

## Data Availability

The original contributions presented in the study are included in the article/**Supplementary Material**. Further inquiries can be directed to the corresponding author.
