# Supplementary material for: Analysis of epidemiology and nomogram construction for prediction and clinical decision-making in gliomas
Source: Front Immunol. 2025 Aug 1;16:1624142. doi: 10.3389/fimmu.2025.1624142 (PMC12354384; doi:10.3389/fimmu.2025.1624142)
Supplement: Supplementary file 1 [file DataSheet1.pdf]

Supplementary Material

Supplementary Figures and Tables

1.1 Supplementary Figures

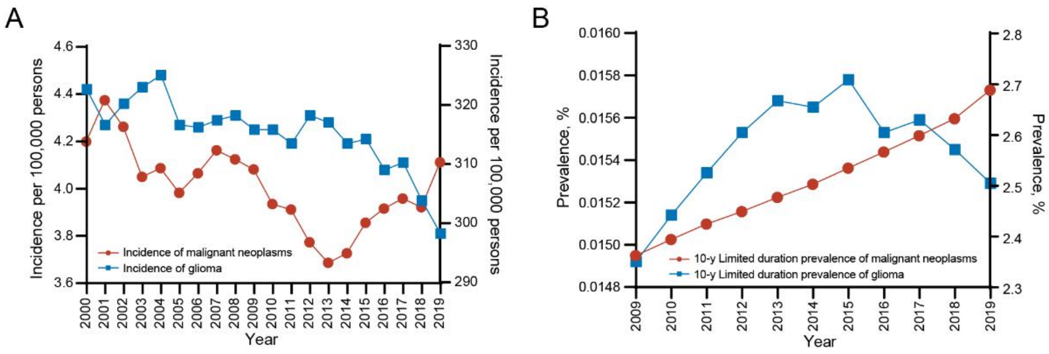

Supplementary Figure 1. Overall Incidence and 10-years Limited Prevalence of Gliomas and Malignant Neoplasms Over Time: A. Overall incidence of gliomas and malignant neoplasms by years (2000-2019). B. 10-years limited prevalence of gliomas and malignant neoplasms by years (2009-2019).

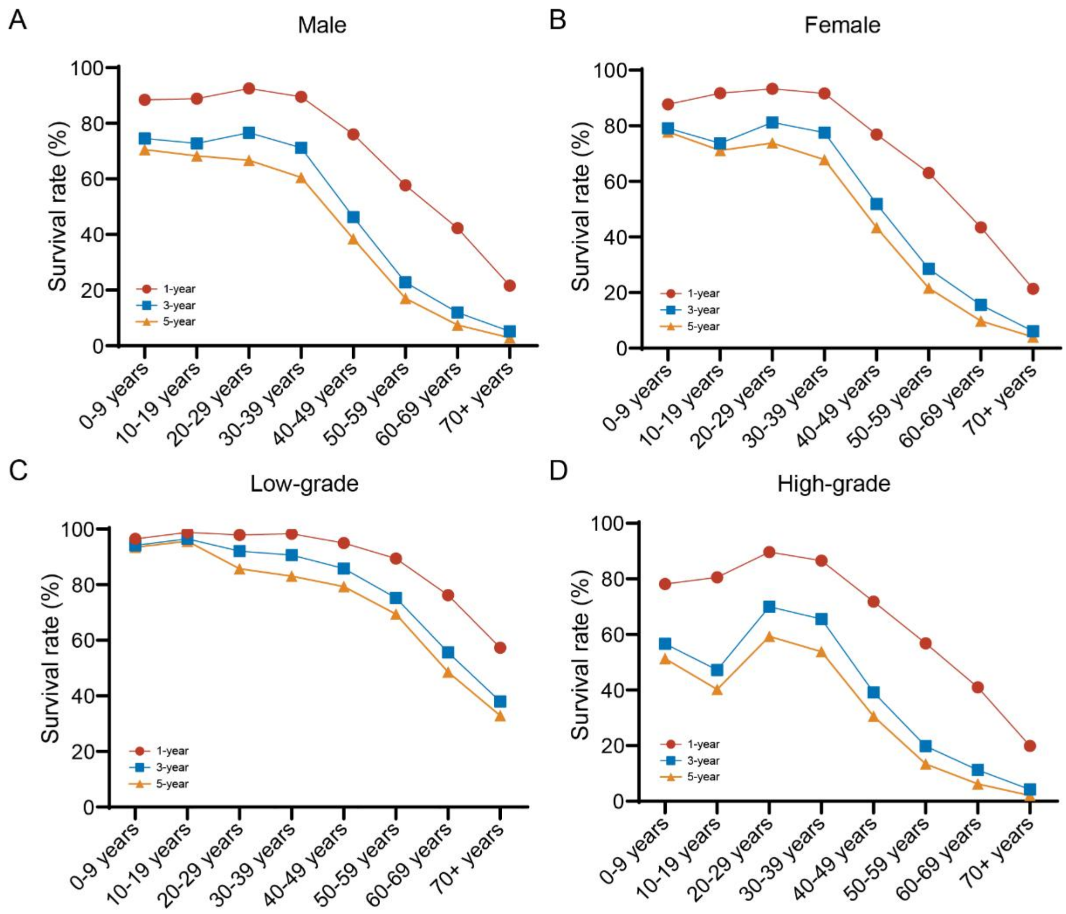

Supplementary Figure 2. Comparison of 1-/3-/5-year relative survival for glioma by sex and grade groups. Age-specific 1-/3-/5-year relative survival for (A) male, (B) female, (C) low-grade gliomas, and (D) high-grade gliomas.

1.2 Supplementary Tables

Supplementary Table 1. Histological ICD codes used to identify glioma patients from SEER

CBTRUS histologies	ICD-O-3 Histology codes
Glioblastoma	9440/3, 9441/3, 9442/3
Diffuse astrocytoma	9400/3, 9410/3, 9411/3, 9420/3
Anaplastic astrocytoma	9401/3
Oligodendroglioma	9450/3
Anaplastic oligodendroglioma	9451/3, 9460/3
Ependymal tumors	9391/3, 9392/3, 9393/3
Glioma malignant, not otherwise specified (NOS)	9380/3
Oligoastrocytic tumors	9382/3
Pilocytic astrocytoma	9421/1, 9425/3
Unique astrocytoma variants	9381/3, 9424/3
Other neuroepithelial tumors	9423/3, 9430/3

ICD-O-3, The International Classification of Diseases for Oncology, Third Edition, and site codes; SEER, The Surveillance, Epidemiology, and End Results; CBTRUS, The Central Brain Tumor Registry of the United States.

Supplementary Table 2. Site codes for glioma patients from SEER

Site	ICD-O-3 Site Code
**Cerebrum**	C71.0
Frontal lobe of brain	C71.1
Temporal lobe of brain	C71.2
Parietal lobe of brain	C71.3
Occipital lobe of brain	C71.4
**Ventricle**	C71.5
**Cerebellum**	C71.6
**Brain stem**	C71.7
**Other brain**	C71.8- C71.9
Overlapping lesion of brain	C71.8
Brain, NOS	C71.9
**Spinal cord and cauda equina**	C72.0- C72.1
Spinal cord	C72.0
Cauda equine	C72.1
**Cranial nerves**	C72.2- C72.5
Olfactory nerve	C72.2
Optic nerve	C72.3
Acoustic nerve	C72.4

Cranial nerve, NOS	C72.5
**Other nervous system**	C72.8- C72.9
Overlapping lesion of brain and central nervous system	C72.8
Nervous system, NOS	C72.9
**Meninges (cerebral & spinal)**	C70.0- C70.9
Cerebral meninges	C70.0
Spinal meninges	C70.1
Meninges, NOS	C70.9
**Pituitary and craniopharyngeal duct**	C75.1- C75.2
Pituitary gland	C75.1
Craniopharyngeal duct	C75.2
**Pineal gland**	C75.3
**Olfactory tumors of the nasal cavity**	C30.0

ICD-O-3, The International Classification of Diseases for Oncology, Third Edition, and site codes; SEER, The Surveillance, Epidemiology, and End Results.

Supplementary Table 3. Baseline clinicopathological characteristics of glioma

Characteristic	N	%
**Age, y**		
≤ 39	17,911	25.21
40-49	9,165	12.90
50-59	14,410	20.28
60-69	15,125	21.29
≥ 70	14,429	20.31
**Sex**		
Male	40,500	57.01
Female	30,540	42.99
**Race**		
Non-Hispanic White	52,443	73.82
Non-Hispanic Black	4,299	6.05
Non-Hispanic AI/AN	358	0.50
Non-Hispanic Asian/PI	3,845	5.41
Hispanic	9,816	13.82
Unknown	279	0.39
**Household income**		
< $35,000	896	1.26

$35,000-$54,999	13,132	18.49
$55,000-$74,999	33,523	47.19
≥ $75,000	23,483	33.06
Unknown	6	0.01
**Urban/rural**		
Urban	62,691	88.25
Rural	8,287	11.67
Unknown	62	0.09
**Marital status**		
Married	39,189	55.16
Single/Sep/Div/Wid	29,348	41.31
Unknown	2,503	3.52
**Size (mm)**		
≤ 20	5,573	7.84
21-40	18,715	26.34
≥ 41	29,192	41.09
Unknown	17,560	24.72
**Histology**		
Anaplastic astrocytoma	5,382	7.58
Diffuse astrocytoma	6,654	9.37

Glioblastoma	41,125	57.89
Oligodendroglioma	3,532	4.97
Other gliomas	14,347	20.20
**Grade**		
Low-grade	5,590	7.87
High-grade	22,447	31.60
Unknown	43,003	60.53
**Primary tumor sites**		
Cerebrum	49,307	69.41
Ventricle	1,070	1.51
Cerebellum	2,578	3.63
Brain stem	2,075	2.92
Spinal cord and cauda equina	2,187	3.08
Cranial nerves	246	0.35
Other brain and nervous system	13,577	19.11

Non−Hispanic AI/AN, Non-Hispanic American Indian/Alaska Native; Non−Hispanic Asian/PI, Non-Hispanic Asian or Pacific Islander; Single/Sep/Div/Wid, Single/Separated/Divorced/Widowed.

Supplementary Table 4. Incidence of glioma over time

**Registry**	**Year**	**Rate**	**Lower CI**	**Upper CI**
SEER 17	2000	4.42	4.27	4.57
	2001	4.27	4.12	4.42
	2002	4.36	4.22	4.52
	2003	4.43	4.28	4.59
	2004	4.49	4.34	4.64
	2005	4.28	4.14	4.43
	2006	4.26	4.12	4.41
	2007	4.3	4.15	4.45
	2008	4.31	4.17	4.46
	2009	4.25	4.11	4.4
	2010	4.26	4.12	4.4
	2011	4.2	4.06	4.34
	2012	4.32	4.18	4.47
	2013	4.28	4.14	4.42
	2014	4.2	4.06	4.34
	2015	4.23	4.09	4.37
	2016	4.09	3.96	4.22
	2017	4.11	3.98	4.25

	2018	3.95	3.82	4.08
	2019	3.81	3.68	3.94

CI, confidence interval.

Supplementary Table 6. 10-year prevalence of glioma

Year	10-year duration Prevalence	10-year Count	Population at Prevalence Date
2009	0.01492%	12,216.10	81,902,989.00
2010	0.01514%	12,510.90	82,607,648.00
2011	0.01534%	12,768.00	83,237,422.00
2012	0.01553%	13,018.00	83,827,958.00
2013	0.01568%	13,235.40	84,415,829.00
2014	0.01565%	13,307.50	85,019,798.50
2015	0.01578%	13,505.10	85,608,158.00
2016	0.01553%	13,374.10	86,130,300.50
2017	0.01559%	13,490.00	86,532,157.50
2018	0.01545%	13,413.00	86,804,431.00
2019	0.01529%	13,291.9	86,956,440.00

Supplementary Table 9. The risk tables to Kaplan-Meier plots.

	1 year			3 year			5 year		
	Number at risk at start of study	Numb er of deaths	Cumulative proportion surviving (SE)	Numbe r at risk at start of study	Numb er of deaths	Cumulative proportion surviving (SE)	Number at risk at start of study	Numb er of deaths	Cumulative proportion surviving (SE)
0-9 years	782	107	0.881 (0.11)	619	205	0.768 (0.14)	529	226	0.741 (0.15)
10-19 years	683	75	0.902 (0.011)	518	202	0.732 (0.016)	433	226	0.696 (0.017)
20-29 years	1221	95	0.929 (0.007)	959	278	0.787 (0.011)	747	379	0.699 (0.013)
30-39 years	1800	194	0.904 (0.007)	1331	517	0.738 (0.010)	1006	692	0.635 (0.011)
40-49 years	2117	663	0.764 (0.008)	1237	1421	0.485 (0.010)	898	1617	0.404 (0.010)
50-59 years	2312	1573	0.599 (0.008)	865	2885	0.251 (0.007)	545	3086	0.189 (0.007)
60-69 years	1596	2184	0.427 (0.008)	422	3244	0.134 (0.006)	215	3390	0.084 (0.005)
70+ years	709	2764	0.215 (0.007)	159	3273	0.056 (0.004)	72	3330	0.034 (0.003)

SE, Standard error.

Supplementary Table 10. Baseline clinicopathological characteristics of patients in the training and validation cohorts

Variables	Training set N (%)	Validation set N (%)	Odds ratio (95% CI)
**Age, y**			
≤ 39	3229 (26.36%)	1855 (25.55%)	1.00
40-49	1910 (15.59%)	935 (12.88%)	0.85 (0.77-0.94)
50-59	2512 (20.50%)	1482 (20.41%)	1.03 (0.94-1.12)
60-69	2247 (18.34%)	1657 (22.82%)	1.28 (1.18-1.40)
≥ 70	2353 (19.21%)	1332 (18.34%)	0.99 (0.90-1.08)
**Sex**			
Male	7036 (57.43%)	4122 (56.77%)	1.00
Female	5215 (42.57%)	3139 (43.23%)	1.03 (0.97-1.09)
**Race**			
Non-Hispanic White	9232 (75.36%)	5210 (71.75%)	1.00
Non-Hispanic Black	696 (5.68%)	436 (6.00%)	1.11 (0.98-1.26)
Non-Hispanic AI/AN	48 (0.39%)	33 (0.45%)	1.22 (0.78-1.90)
Non-Hispanic Asian/PI	594 (4.85%)	443 (6.10%)	1.32 (1.16-1.50)
Hispanic	1681 (13.72%)	1139 (15.69%)	1.20 (1.11-1.30)
**Marital status**			
Single	2996 (24.46%)	2053 (28.27%)	1.00

Married	7286 (59.47%)	4204 (57.90%)	0.84 (0.79-0.90)
Sep/Div/Wid	1969 (16.07%)	1004 (13.83%)	0.74 (0.68-0.82)
**Household income**			
< $35,000	93 (0.76%)	95 (1.31%)	1.00
$35,000-$54,999	2191 (17.88%)	1629 (22.43%)	0.73 (0.54-0.98)
$55,000-$74,999	6419 (52.40%)	3562 (49.06%)	0.54 (0.41-0.73)
≥ $75,000	3548 (28.96%)	1975 (27.20%)	0.54 (0.41-0.73)
**Urban/rural**			
Rural	1628 (13.29%)	886 (12.20%)	1.00
Urban	10623 (86.71%)	6375 (87.80%)	1.10 (1.01-1.20)
**Size (mm)**			
≤ 20	1345 (10.98%)	760 (10.47%)	1.00
21-40	4377 (35.73%)	2442 (33.63%)	0.99 (0.89-1.09)
≥ 41	6529 (53.29%)	4059 (55.90%)	1.10 (1.00-1.21)
**Grade**			
Low-grade	2430 (19.84%)	955 (13.15%)	1.00
High-grade	9821 (80.16%)	6306 (86.85%)	1.63 (1.51-1.77)
**Primary tumor sites**			
Brain stem	296 (2.42%)	185 (2.55%)	1.00

Cerebellum	322 (2.63%)	188 (2.59%)	0.93 (0.72-1.21)
Cerebrum	9042 (73.81%)	5438 (74.89%)	0.96 (0.80-1.16)
Spinal cord and cauda equina	186 (1.52%)	106 (1.46%)	0.91 (0.67-1.23)
Ventricle	159 (1.30%)	109 (1.50%)	1.10 (0.81-1.49)
Other brain and nervous system	2246 (18.33%)	1235 (17.01%)	0.88 (0.72-1.07)
**Histology**			
Anaplastic astrocytoma	1588 (12.96%)	1474 (20.30%)	1.00
Diffuse astrocytoma	1497 (12.22%)	537 (7.40%)	0.39 (0.34-0.44)
Glioblastoma	6041 (49.31%)	3598 (49.55%)	0.64 (0.59-0.70)
Oligodendroglioma	618 (5.04%)	194 (2.67%)	0.34 (0.28-0.40)
Other gliomas	2507 (20.46%)	1458 (20.08%)	0.63 (0.57-0.69)
**Chemotherapy**			
No	6153 (50.22%)	2362 (32.53%)	1.00
Yes	6098 (49.78%)	4899 (67.47%)	2.09 (1.97-2.22)
**Radiation**			
None	3992 (32.59%)	2040 (28.10%)	1.00
External beam radiation	8259 (67.41%)	5221 (71.90%)	1.24 (1.16-1.32)
**Surgery**			
No surgery	2646 (21.60%)	1316 (18.12%)	1.00
Biopsy/subtotal resection	5573 (45.49%)	3215 (44.28%)	1.16 (1.07-1.26)

Gross total resection	4032 (32.91%)	2730 (37.60%)	1.36 (1.25-1.48)

CI, confidence interval; Non−Hispanic AI/AN, Non-Hispanic American Indian/Alaska Native; Non−Hispanic Asian/PI, Non-Hispanic Asian or Pacific Islander; Sep/Div/Wid, Separated/Divorced/Widowed.

Supplementary Table 11. Detailed score assignment for specific number/category of the parameters included in the nomogram

Prognostic Variable	Category	Score
Age (year)	⩽ 39	0
	40-44	19
	45-49	33
	50-54	48
	55-59	57
	60-64	66
	65-69	78
	⩾ 70	100
Site	Spinal cord	0
	Cerebellum	12
	Ventricle	42
	Brain stem	46
	Cerebrum	47
	Other	56
Tumor grade	Low grade	0
	High grade	80
Histology	Other gliomas	0
	Oligodendrogliomal	7
	Anaplastic astrocytoma	35
	Diffuse astrocytoma	45
	Glioblastoma	77
Surgery	Gross total resection	0
	Biopsy/subtotal resection	18
	No	50
Radiation	External beam radiation	0

	No	22
Chemotherapy	Yes	0
	No	23
1-year survival<br>Total score	1-year survival	
	0.01	394
	0.1	349
	0.2	326
	0.3	308
	0.4	290
	0.5	272
	0.6	252
	0.7	229
	0.8	199
	0.9	151
	0.95	104
3-year survival<br>Total score	3-year survival	
	0.01	330
	0.1	286
	0.2	263
	0.3	244
	0.4	226
	0.5	208
	0.6	189
	0.7	166
	0.8	135
	0.9	87

	0.95	41
5-year survival<br>Total score	5-year survival [illegible]	
	0.01	313
	0.1	268
	0.2	245
	0.3	226
	0.4	209
	0.5	191
	0.6	171
	0.7	148
	0.8	118
	0.9	70
	0.95	23

R code for nomogram development

```
library(survival)
library(rms)
glioma <- read.csv('training.csv')
glioma <- as.data.frame(glioma)
head(glioma)
glioma$censor <- ifelse(glioma$fustat=='Dead', 1, 0)
dd<-datadist(glioma)
options(datadist='dd')
coxm1 <- cph(Surv(futime,
   censor==1)~Age+Site+Grade+Histology+Surgery+Radiation+Chemotherapy, x=T, y=T, data=
   glioma, surv=T)
summary(coxm1)
surv <- Survival(coxm1)
surv1 <- function(x)surv(1*12, lp=x)
surv2 <- function(x)surv(1*36, lp=x)
surv3 <- function(x)surv(1*60, lp=x)
nom1<-nomogram(coxm1, fun=list(surv1, surv2, surv3), lp= F,funlabel=c('1-Year survival
   probability', '3-Year survival probability', '5-Year survival probability'), maxscale=100,
   fun.at=c("0.95", '0.9', '0.80', '0.70', '0.6', '0.5', '0.4', '0.3', '0.2', '0.1', '0.01'))
plot(nom1, xfrac=.30)
```
